# The correlation of metabolic and renal biomarkers with vitamin D status in postmenopausal women

**DOI:** 10.5937/jomb0-41044

**Published:** 2023-10-27

**Authors:** Aleksandra Klisić, Irena Radoman-Vujačić, Jelena Kostadinović, Ana Ninić

**Affiliations:** 1 University of Montenegro, Faculty of Medicine, Primary Health Care Center, Podgorica, Montenegro; 2 University of Montenegro - Faculty of Medicine, Clinical Center of Montenegro, Department of Internal medicine, Podgorica, Montenegro; 3 Clinical Hospital Center Bežanijska kosa, Belgrade; 4 University of Belgrade, Faculty of Pharmacy, Department for Medical Biochemistry, Belgrade

**Keywords:** postmenopausal, vitamin D deficiency, cardiometabolic risk, inflammation, obesity, žene u postmenopauzi, deficit vitamina D, kardiometabolički rizik, inflamacija, gojaznost

## Abstract

**Background:**

To our knowledge, the mutual involvement of a variety of metabolic and renal biomarkers and vitamin D (determined as 25-hydroxyvitamin D [25(OH)D]) in postmenopausal women has not been examined yet. Therefore, we aimed to explore such a relationship by a thorough statistical multimarker approach.

**Methods:**

A total of 150 (diabetes and cardiovascular disease-free) postmenopausal women were included. Anthropometric and biochemical parameters were measured. The fatty liver index (FLI) and Homeostasis model assessment of insulin resistance (HOMA-IR) were calculated. Univariate and multivariate binary logistic regression analyses were used to test the predictions of cardiometabolic markers for [25(OH)D] status. Principal component analysis (PCA) was applied to explore the effect of examined biomarkers on [25(OH)D] status.

## Abstract (sr)

**Uvod:**

Prema na{em saznanju zajednički uticaj različitih metaboličkih i renalnih biomarkera na nivo vitamin D (određenog kao 25-hidroksivitamin D [25(OH)D]) kod žena u postmenopauzi još{ uvijek nije ispitivan. Stoga je cilj istraživanja bio da se ispita povezanost ovih biomarkera primenom temeljne statističke analize koja uključuje više navedenih biomarkera.

**Metode:**

Ukupno 150 žena u postmenopauzi (koje nemaju dijabetes, ni kardiovaskularna oboljenja) je uključeno u istraživanje. Mereni su antropometrijski i biohemijski parametri. Indeks masne jetre (FLI) i indeks insulinske rezistenci je (HOMA-IR) su izračunati. Univarijantna i multivarijantna binarna logistička regresija su primenjene da bi se odredili kardiometabolički prediktori nivoa [25(OH)D].

**Rezultati:**

Univarijantna binarna regresiona analiza je pokazala pozitivnu korelaciju između FLI i [25(OH)D] [OR=1.036 (1.020-1.052), P<0,001] i HOMA-IR sa [25(OH)D] [OR=2.385 (1.601-3.551), P<0,001]. Multivarijantna logistička regresiona analiza je pokazala najbolji model sa 2 markera, tj. FLI [OR=1.032 (1.016-1.048), P<0,001] i glukoza [OR=2.167 (1.001-4.693), P=0,050] koji su korelirali sa prvom tercilom vrednosti [25(OH)D]. PCA je izdvojila 2 značajna faktora: Renal function-related faktor i Metabolic syndrome-related faktor. Renal function-related faktor [tj. kreatinin, mokraćna kiselina, retinol-vezujući protein 4 (RBP4) i cistatin C] su objasnili 29% varijanse. Takođe, Metabolic syndromerelated faktor (tj. HOMA-IR, FLI, hsCRP i fibrinogen) su objasnili 29% varijanse ispitivanih parametara. Štaviše, binarna logistička regresiona analiza je pokazala pozitivnu prediktivnu sposobnost oba faktora, tj. Renal functionrelated faktora i Metabolic syndrome-related faktora (P=0,044 i P<0,001) za nivo [25(OH)D].

**Zaključak:**

Zajedničko dejstvo renalnih biomarkera i markera metaboličkog sindroma (tj. insulinska rezistencija, inflamacija i nealkoholna steatoza jetre) mogu diferencirati žene u postmenopauzi sa nižim nivoom [25(OH)D] u odnosu na ostale žene.

## Introduction

It is established that vitamin D (obtained from a diet or through the exposure of the skin to the sun) is metabolized by two important steps of hydroxylation, i.e. in the liver (25-hydroxyvitamin D [25(OH)D]) and in the kidney (1,25-dihydroxyvitamin D [1,25(OH)2D]) [1]. The major form of vitamin D in circulation is 25-hydroxyvitamin D (25[OH]D) [1].

In recent decades, great attention has been paid to the examination of the vitamin D level in a variety of inflammatory disorders in the human population, such as obesity, polycystic ovary syndrome, type 2 diabetes mellitus, non-alcoholic fatty liver disease (NAFLD), etc. [2] [3] [4] [5] [6].

However, despite the great number of investigations related to this topic, the findings are still inconclusive. While some cross-sectional studies showed an inverse correlation between glucose homeostasis indices and other metabolic syndrome components, as well as inflammation parameters [2] [7] with vitamin D, the others did not [8] [9] [10]. Similarly, discordant results were shown regarding the effect of vitamin D supplementation on cardiometabolic risk factors [3] [11] [12] [13] [14] [15].

Compared to women of reproductive age, women who experience postmenopause are faced with hormonal changes due to estrogen deficiency [16] [17]. Moreover, metabolic disturbances such as insulin resistance due to re-distributed adipose tissue towards visceral compartments with concomitant central obesity, dyslipidemia and hypertension (all of which are well-known components of metabolic syndrome) are present in postmenopausal period. Liver steatosis as a hepatic manifestation of metabolic syndrome is highly prevalent, as well [16]. Recently, the association between NAFLD and chronic kidney disease (CKD) with vitamin D level has also been confirmed due to their common metabolic pathways [18] [19].

As far as we know, the joint involvement of metabolic and renal biomarkers and vitamin D (determined as [25(OH)D]) in postmenopausal women has not been investigated yet. Since that vitamin D becomes biotransformed in the liver and the kidney, it would be expected that diseases that affect the liver/kidney might influence the level of vitamin D in circulation. If such is the case, vitamin D supplementation might play a beneficial role in the prevention and/or delay of such cardiometabolic disorders in postmenopausal women.

Therefore, we aimed to examine a relationship of metabolic and renal biomarkers with [25(OH)D]) in postmenopausal women by a thorough statistical multimarker approach called Principal Component Analysis (PCA), as reported in our previous studies related to cardiometabolic disorders [20] [21] [22].

## Materials and methods

### Study population

After obtaining the approval of the Institutional Ethics Committee and written informed consent of the participants, and following the principles of the Declaration of Helsinki, the research included consecutively a cohort of 150 postmenopausal women free of known cardiometabolic diseases.

Each woman who voluntarily accepted participation in the study was given a questionnaire that included demographic data, history of diseases, medications use, smoking and alcohol consumption history. On the same day clinical examinations (i.e. anthropometric and blood pressure measurements) were provided, as described previously [16].

Women were included in the study if reported to be postmenopausal (i.e., if reported the absence of menstruation for more than 1 year), with no using any medications or hormone replacement therapies, with no history of cardiometabolic, malignant and autoimmune diseases.

Exclusion criteria were as follows: smoking, ethanol consumption >20 g/day, high sensitivity C-reactive protein (hsCRP) ≥10 mg/L, diabetes mellitus, liver disease other than steatosis, hyperthyroidism, hypothyroidism, cardiovascular and kidney diseases and medications used in the last 6 months. Also, women that reported irregular menstrual bleeding for less than 1 year or premenopausal women were excluded from the research.

Non-alcoholic fatty liver disease is assessed by fatty liver index (FLI), an algorithm based on body mass index (BMI), waist circumference (WC), triglycerides (TG) and gamma-glutamyl transferase (GGT), as the best validated score of hepatic steatosis in the general population [23].

### Biochemical analyses

The blood samples were provided in the morning after at least 8 hours of an overnight fast. After blood collection the samples were left to clot for 30 minutes and centrifuged at 3000 rpm for 10 minutes. Fasting glucose was measured immediately whereas the other obtained sera samples were aliquoted and kept at -80°C, until analyses of total cholesterol (TC), high density lipoprotein cholesterol (HDL-c), low density lipoprotein cholesterol (LDL-c), TG, aspartate aminotransferase (AST), alanine aminotransferase (ALT), GGT, uric acid and creatinine. All these parameters were determined using standardized enzymatic procedures, spectrophotometrically on Roche Cobas 400, Mannheim, Germany.

Insulin levels were determined by chemiluminescent immunometric assay (Immulite 2000, Siemens, Muenchen, Germany). HOMA-IR was calculated: HOMA-IR=Fasting glucose (mmol/L) x fasting insulin (μIU/L)/22.5 [16].

Retinol-binding protein 4 (RBP4), hsCRP and cystatin C levels were measured with a nephelometric assay (Behring Nephelometer Analyzer, Marburg, Germany).

The levels of serum [25(OH)D] were determined by electrochemiluminescence (Cobas 6000/e601, Roche Diagnostics, Mannheim, Germany). Although, the whole population of patients had low vitamin D according to references values (less than 75 nmol/L) we have arbitrary decided to present the first tertile of the vitamin D values (less than 37.19 nmol/L) as risky and compared it with the other two tertiles.

### Statistical analysis

Statistical software SPSS version 21.0 (SPSS Inc., Chicago, USA) was used for statistical analysis. Two-tailed P values less than 0.05 were considered significant.

Variables with Gaussian distribution were shown as mean ± standard deviation and compared by the Student *t*-test. Variables that did not follow Gaussian distribution were shown as median (interquartile range) and compared by the Mann-Whitney *U*-test. The correlation of [25(OH)D] and other clinical data were tested by the Spearman's correlation analysis and the results were given as a correlation coefficient (ρ). The associations of [25(OH)D] with FLI and HOMA-IR/glucose were assessed with univariate and multivariate binary logistic regression analysis with backward selection criterion. Data from these analyses were given as odds ratio (OR) and 95% Confidence Interval (CI). The explained variation in [25(OH)D] levels was given by Nagelkerke R^2^ value. Further data analysis involved Principal Component Analysis (PCA) with varimax rotation to reduce the number of examined variables to a smaller number of factors. Eigenvalues greater than 1.0 was used as a criterion to determine which factor should be extracted. Markers with factor loadings greater and equal to 0.5 were used for factor interpretation. PCA allowed us to calculate scores for factors. Scores were included as the independent variables in the following binary logistic regression analysis to determine potential significant predictors for lower [25(OH)D] levels.

## Results

Table 1 shows the clinical characteristics of postmenopausal women. BMI, WC, systolic blood pressure (SBP), diastolic blood pressure (DBP), TG, glucose, insulin, HOMA-IR, uric acid, cystatin C, hsCRP, fibrinogen and FLI were higher in women in the 1. tertile of [25(OH)D] levels than women in the 2. and 3. tertile of [25(OH)D] levels. On the contrary, only HDL-c levels were lower in women in the 1. tertile of [25(OH)D] levels.

**Table 1:** General characteristics of tested population Normally distributed variables are presented as mean ± standard deviation and compared by the Student *t*-test. * Skewed distributed variables are presented as median and interquartile range and compared by the Mann-Whitney *U*-test. Categorical variables are presented as absolute frequencies.

Variable	1. tertile of [25(OH)D]<br>levels < 37.19 nmol/L	2. and 3. tertile of<br>[25(OH)D] levels ≥ 37.19 nmol/L	P
Postmenopausal women, N	50	100	
Age, years	56±5	57±5	0.593
BMI, kg/m^2^	28.6±4.5	25.4±3.8	<0.001
WC, cm*	98 (88-103)	86 (78–94)	<0.001
SBP, mmHg*	139 (120–155)	126 (107–145)	0.005
DBP, mmHg*	90 (84–95)	82 (70–94)	0.002
TC, mmol/L	6.4±1.1	6.5±1.0	0.488
HDL-c, mmol/L	1.6±0.4	1.8±0.4	0.009
LDL-c, mmol/L	4.3±1.0	4.3±1.0	0.804
TG, mmol/L*	1.4 (1.1–2.3)	1.2 (0.9–1.7)	0.039
Glucose, mmol/L*	5.4 (5.1–6.0)	5.2 (5.0–5.5	0.005
Insulin, μIU/L*	8.4 (6.2-12.0)	5.5 (4.2–7.6)	<0.001
HOMA-IR*	2.0 (1.5–3.1)	1.2 (1.0–1.8)	<0.001
GGT, IU/L*	13 (9–19)	11 (9–14)	0.055
Total bilirubin, μmol/L*	7.1 (6.0–8.5)	7.8 (6.4–10.4)	0.160
Uric acid, μmol/L	286±62	250±63	0.001
Creatinine, μmol/L	56±6	55±7	0.431
Cystatin C, μmol/L	0.80±0.10	0.75±0.10	0.002
RBP4, g/L	42.08±8.67	40.46±9.24	0.303
HsCRP, mg/L*	1.3 (0.8–2.4)	0.7 (0.4–1.7)	0.002
Fibrinogen, g/L*	3.9 (3.5–4.4)	3.6 (3.3–4.1)	0.045
FLI*	41 (23–71)	21 (8–37)	<0.001
[25(OH)D], nmol/L	29 (23–34)	48 (41–58)	<0.001

Further, we wanted to determine the correlation between [25(OH)D] levels and examined markers in all women (Table 2). Spearman's correlation analysis demonstrated a significant negative correlation between [25(OH)D] and BMI, WC, SBP, DBP, TG, glucose, insulin, HOMA-IR, uric acid, cystatin C, hsCRP, fibrinogen and FLI. [25(OH)D] correlated positively with HDL-c.

**Table 2:** Spearman’s correlation coefficients of [25(OH)D] and clinical data Data are given as coefficients of correlation Rho (ρ)

Variable	ρ	P
Age, years	0.020	0.811
BMI, kg/m^2^	-0.377	<0.001
WC, cm	-0.379	<0.001
SBP, mmHg	-0.257	0.002
DBP, mmHg	-0.236	0.004
TC, mmol/L	0.016	0.850
HDL-c, mmol/L	0.189	0.021
LDL-c, mmol/L	-0.062	0.454
TG, mmol/L	-0.197	0.016
Glucose, mmol/L	-0.252	0.002
Insulin, μIU/L	-0.389	<0.001
HOMA-IR	-0.423	<0.001
GGT, IU/L	-0.125	0.126
Total bilirubin, μmol/L	0.062	0.448
Uric acid, μmol/L	-0.285	<0.001
Creatinine, μmol/L	-0.043	0.600
Cystatin C, μmol/L	-0.224	0.006
RBP4, g/L	-0.120	0.144
HsCRP, mg/L	-0.272	0.001
Fibrinogen, g/L	-0.184	0.024
FLI	-0.383	<0.001

Our further intention was to search for in-depth associations of [25(OH)D] with FLI and HOMA-IR. Therefore, we conducted a binary logistic regression analysis (Table 3). Both variables (i.e., FLI and HOMA-IR) were significantly positively associated with [25(OH)D] levels.

**Table 3:** Odds ratios (OR) after univariate and multivariate binary logistic regression analysis with backward selection in the best-fit model for FLI and HOMA-IR/glucose predictingabilities towards the first tertile of [25(OH)D] values, i.e. lower [25(OH)D] levels

Predictors	◻β (SE)	Wald	Unadjusted<br>OR (95%CI)	P	Nagelkerke<br>R^2^
FLI	0.075<br>(0.008)	21.099	1.036<br>(1.020–1.052)	<0.001	0.211
HOMA-IR	0.869<br>(0.203)	18.305	2.385<br>(1.601–3.551)	<0.001	0.200
Model 1	◻β (SE)	Wald	AdjustedOR<br>(95%CI)	P	Nagelkerke<br>R^2^
FLI	0.032<br>(0.008)	15.968	1.032<br>(1.016–1.048)	<0.001	0.242
Glucose	0.773<br>(0.394)	3.849	2.167<br>(1.001–4.693)	0.050	

Univariate binary regression analysis revealed positive associations of FLI and HOMA-IR with [25(OH)D] demonstrated by following OR 1.036 and 2.385, respectively. Furthermore, the multivariate logistic regression analysis with backward selection enabled us to find the best model consisting of two markers, i.e., FLI (P<0.001) and glucose (P=0.05) associated with the first tertile of [25(OH)D]. Adjusted R^2^ for the best model was 0.242, which means that 24.2% of variation in lower [25(OH)D] values could be explained by this model. On the contrary, HOMA-IR was found to be the single marker for lower [25(OH)D], because backward selection did not segregate any best model for lower [25(OH)D].

PCA was applied to biochemical data to find their relationship with [25(OH)D] status. Sample adequacy was confirmed with the Keiser-Meier-Olkin measure (KMO index = 0.783). Bartlett's test of sphericity was significant (P<0.001). After initial analysis total bilirubin was excluded because it was the only marker of factor 3. The factors were given in Table 4. This PCA extracted 2 significant factors with a total percent of the explainable variation of 58% of the investigated parameters. The Renal function-related factor explained 29% of the variance and it was associated with positive loading of creatinine, RBP4, cystatin C and uric acid. The Metabolic syndrome-related factor explained 29% of the variance and it was associated with positive loading of hsCRP, HOMA-IR, FLI and fibrinogen. By using scores derived from PCA, a univariate binary logistic regression analysis was conducted. We demonstrated the positive predictive ability of both factors for the first tertile of [25(OH)D] levels, i.e. lower [25(OH)D] levels. The Renal function-related factor was positively associated with the first tertile of [25(OH)D] levels [OR=1.455 (1.009–2.056), P=0.044]. Increased Renal function-related marker values were associated with a 1.455 greater probability for lower [25(OH)D] levels. This first factor explained variation in [25(OH)D] levels by 3.9% which was demonstrated by Nagelkerke R^2^ of 0.039. As well, the Metabolic syndrome-related factor was positively associated with the first tertile of [25(OH)D] levels [OR=2.273 (1.536–3.362), P<0.001]. Increased Metabolic syndrome-related factor values were associated with a 2.273 greater probability for lower [25(OH)D] levels. The second factor explained variation in [25(OH)D] levels by 17.4% which was demonstrated by Nagel-kerke R^2^=0.174.

**Table 4:** Factors extracted by principal component analysis with percent of variability and variables’ loadings in relationship with [25(OH)D] levels

Factors	Variables(loadings)	Factorvariability
Renal function-related<br>factor	Creatinine (0.769)<br>RBP4 (0.706)<br>Cystatin C (0.695)<br>Uric acid (0.660)	29%
Metabolic<br>syndrome-related<br>factor	HsCRP (0.799)<br>HOMA-IR (0.746)<br>FLI (0.700)<br>Fibrinogen (0.515)	29%

## Discussion

This study is the first that investigated the involvement of a variety of cardiometabolic biomarkers in relation to [25(OH)D] status among diabetes-free and cardiovascular-free postmenopausal women. Moreover, this study is the first to apply a thorough statistical approach, such as PCA to gain deeper insight into the relationship between [25(OH)D] status and metabolic and kidney biomarkers.

We have shown the relationship between lower serum [25(OH)D] and HOMA-IR and FLI (as a proxy of NAFLD), respectively. We have also reported that increased Renal function-related marker values and Metabolic syndrome-related factor values were associated with a 1.455 and 2.273 greater probability of lower [25(OH)D] levels, respectively.

Previous reports have shown conflicting findings regarding the association between low serum vitamin D levels and NAFLD [5] [24] [25]. The possible discrepancies between studies might in part be attributed to differences in diagnostic procedures of NAFLD, differences in sample size, variety of associated comorbidities, as well as different ages and ethnicities of examined populations.

The associations between vitamin D deficiency and NAFLD, as well as with CKD might in part be explained by the process of hydroxylation of vitamin D that occurs both in the liver and the kidney [26]. Those findings suggest that vitamin D deficiency may be one of the links between NAFLD and CKD since these two diseases are interrelated and share common metabolic pathways [18] [19].

Zhang et al. [5] have shown that vitamin D supplementation led to the improvement of insulin resistance and liver steatosis through the process of vitamin D receptor upregulation and hepatocyte nuclear factor 4 overexpression.

We have recently shown the anti-inflammatory and antioxidative properties of vitamin D [27], as well as a decrease in the level of glycated hemoglobin after 3 and after 6 months of vitamin D supplementation in patients with type 2 diabetes mellitus, although the HOMA-IR did not significantly change during the follow-up period [4].

The properties of vitamin D in insulin-sensitivity modulation were recently confirmed by the inhibition of the expression of peroxisome proliferator-activated receptor ϒ (PPAR-ϒ) and reducing peripheral insulin resistance [28]. Vitamin D activates its receptors in pancreatic islet -◻cells and favors insulin secretion which further confirms the beneficial properties of vitamin D [29]. It was also found that vitamin D favors glucose transporter 4 (GLUT-4) expression in adipose tissue cells and upregulates insulin receptor substrate-1 (IRS-1), thus enabling the uptake of glucose in the muscle cells [30]. Moreover, vitamin D diminishes inflammation by the reduction of lipid droplets output from the adipose tissue, thus preventing liver steatosis since de novo lipogenesis and fatty acid oxidation in the liver are attenuated [30].

We have also found the association between [25(OH)D] and each metabolic syndrome component. Our results of higher BMI and WC in the first tertile of [25(OH)D] (i.e. lower vitamin D levels) are in accordance with the study of Metheniti et al. [2] who showed low [25(OH)D] levels in extremely obese young females. However, unlike our study, they also found an inverse correlation between [25(OH)D] levels and an adipokine RBP4.

Similarly, discrepant results were shown regarding the association between inflammation markers and serum [25(OH)D] levels [7] [8] [9]. We have shown a negative correlation between hsCRP and fibrinogen, respectively with vitamin D. Some studies also reported an inverse correlation between hsCRP and serum [25(OH)D] levels [7], whereas the others failed to find the mentioned relationship [8] [9].

The anti-inflammatory potential of vitamin D has been confirmed by investigations on cell cultures [31]. The ability of macrophages to secrete proinflammatory cytokines (CRP, Tumor necrosis factoralpha, Intereukin-6), as well as the promotion of monocyte transformation to macrophages, are diminished by vitamin D [31]. Also, vitamin D lowers the level of inflammation in the adipose tissue by attenuating the activation of nuclear factor-ᴋB (NF-ᴋB) and mitogen-activated protein kinase (MAPK) signaling pathways and disabling the transcription of pro-inflammatory genes [32].

We have also reported the inverse association between [25(OH)D] and cystatin C and uric acid, respectively which is in line with one recent study [33], and opposite to the other [34].

Previous investigations have shown the association between vitamin D deficiency and kidney disease progression [35] [36] which may be in part attributed to the renin-angiotensin-aldosterone system (RAAS) by vitamin D [37]. Concomitantly, the downregulation of RAAS could lower blood pressure [38]. One animal study showed that [1,25(OH)2D] favors suppression of the biosynthesis of renin, whereas vitamin D deficiency stimulates its synthesis [37]. Also, the protective renal effects of vitamin D were shown by lowering levels of transforming growth factor-β and SMAD3 proteins, which are related to oxidative stress and fibrosis in kidneys [39]. Derakhshanian et al. reported [40] that besides a reduction in glucose levels and an increase in insulin secretion, higher serum vitamin D levels can prevent diabetic nephropathy by diminishing fructose-6-phosphate aminotransferase (GFAT) synthesis which is the key enzyme of the hexosamine pathway in the kidney tissue.

Jorde et al. [9] did not report a correlation of serum [25(OH)D] levels neither with BMI, nor with hsCRP in nonsmoking females with BMI ≥28 kg/m^2^, presuming that vitamin D has more enhanced properties in the presence of cardiometabolic diseases, when the immune response is stimulated. However, we included in our study normal weight and overweight/obese postmenopausal women free of known cardiometabolic diseases and we reported significant correlation between cardiometabolic biomarkers and serum [25(OH)D] levels, as well as the relationship between HOMA-IR, glucose and FLI, respectively, and serum [25(OH)D] levels. Additionally, the joint involvement of renal function biomarkers and metabolic syndrome-related features in relation to serum [25(OH)D] level in postmenopausal women was confirmed.

It is important to mention several limitations of this study. Namely, its cross-sectional design does not allow us to confirm the causal link between serum [25(OH)D] levels and examined cardiometabolic biomarkers. We were limited for the measurement of the level of sex hormones in postmenopausal women. Moreover, we included in this research only Montenegrin postmenopausal women, thus the obtained results cannot be generalized to other populations and ethnicities. On the other hand, we included a narrow age range of women who were non-smokers, who were not taking any medications or vitamin D supplements in the last several months, and who were diabetes-free and cardiovascular disease-free. Hence, we aimed to limit the potential confounding factors such as hormonal variability, smoking, medications, co-morbidities, and the influence of vitamin D supplements on the serum [25(OH)D] levels [3] [9] [11] [41]. Additionally, we included a variety of biomarkers that may reflect cardiometabolic disturbances in the postmenopausal period of women, as some of the previous studies reported [16] [17] [42], and applied a deeper statistical approach [20] [21] [22] in relation to serum [25(OH)D] status in the examined cohort.

## Conclusion

Ours is the first study that examined the mutual involvement of renal function biomarkers and metabolic syndrome-related features (i.e. inflammation, insulin resistance and non-alcoholic fatty liver disease) in relation to serum [25(OH)D] level in postmenopausal women. The PCA analysis revealed that these two factors (i.e. kidney and metabolic) can differentiate women with higher [25(OH)D] levels from those women with lower [25(OH)D] levels. Future researches are necessary to explore the causal link between chronic kidney disease, non-alcoholic fatty liver disease and [25(OH)D] deficiency in postmenopausal women.

## Dodatak

### Funding

This research was partially funded by a grant from the Ministry of Science, Montenegro and the Ministry of Education, Science and Technological Development, Republic of Serbia through Grant Agreement with University of Belgrade-Faculty of Pharmacy No: 451-03-68/2022-14/200161.

### Conflict of interest statement

All the authors declare that they have no conflict of interest in this work.
